# Ultrastructure and lipid composition of detergent-resistant membranes derived from mammalian sperm and two types of epithelial cells

**DOI:** 10.1007/s00441-015-2272-y

**Published:** 2015-09-16

**Authors:** Renske A. van Gestel, Jos F. Brouwers, Anton Ultee, J. Bernd Helms, Bart M. Gadella

**Affiliations:** Department of Biochemistry and Cell Biology, Faculty of Veterinary Medicine Utrecht University, Yalelaan 2, 3584 CM Utrecht, The Netherlands; Department of Pathology, Faculty of Veterinary Medicine Utrecht University, Utrecht, The Netherlands; Department of Farm Animal Health, Faculty of Veterinary Medicine Utrecht University, Utrecht, The Netherlands

**Keywords:** Detergent-resistant membranes, Ultrastructure, Cholesterol, Glycolipids, Lipid rafts, Phospholipids, Epithelial cell, Sperm

## Abstract

Lipid rafts are micro-domains of ordered lipids (L_o_ phase) in biological membranes. The L_o_ phase of cellular membranes can be isolated from disordered lipids (L_d_ phase) after treatment with 1 % Triton  X-100 at 4 °C in which the L_o_ phase forms the detergent-resistant membrane (DRM) fraction. The lipid composition of DRM derived from Madin-Darby canine kidney (MDCK) cells, McArdle cells and porcine sperm is compared with that of the whole cell. Remarkably, the unsaturation and chain length degree of aliphatic chains attached to phospholipids is virtually the same between DRM and whole cells. Cholesterol and sphingomyelin were enriched in DRMs but to a cell-specific molar ratio. Sulfatides (sphingolipids from MDCK cells) were enriched in the DRM while a seminolipid (an alkylacylglycerolipid from sperm) was depleted from the DRM. Treatment with <5 mM methyl-ß-cyclodextrin (MBCD) caused cholesterol removal from the DRM without affecting the composition and amount of the phospholipid while higher levels disrupted the DRM. The substantial amount of (poly)unsaturated phospholipids in DRMs as well as a low stoichiometric amount of cholesterol suggest that lipid rafts in biological membranes are more fluid and dynamic than previously anticipated. Using negative staining, ultrastructural features of DRM were monitored and in all three cell types the DRMs appeared as multi-lamellar vesicular structures with a similar morphology. The detergent resistance is a result of protein–cholesterol and sphingolipid interactions allowing a relatively passive attraction of phospholipids to maintain the L_o_ phase. For this special issue, the relevance of our findings is discussed in a sperm physiological context.

## Introduction

Lipid rafts are commonly defined as lipid-ordered (L_o_ phase) micro-domains in the membrane that concentrate specific (e.g., signaling) molecules while excluding others (Helms and Zurzolo 2004; Brown 2006; Fan et al. 2010; Lingwood and Simons 2010; Nyholm 2015). They thereby favor specific protein–protein interactions, enhancing the activation or inactivation of signaling cascades (Simons and Toomre 2000; Brown and London 2000; Kusumi et al. 2004; Surma et al. 2012). Lipid rafts have been shown to accumulate lipid-modified proteins like glycosylphosphatidylinositol (GPI)-anchored proteins (in the outer leaflet) and doubly acylated tyrosine kinases of the Src family (in the inner leaflet) (Simons and Toomre 2000). Lipid rafts have been demonstrated in other subcellular organelles than plasma membranes including endosomes, phagosomes, mitochondria and Golgi membranes (Dermine et al. 2001; Gkantiragas et al. 2001; Gruenberg 2001; Surma et al. 2012; Garofalo et al. 2015). Lipid rafts are enriched in cholesterol and sphingolipids and glycolipids such as gangliosides (Chen et al. 2008) and the forces driving the formation of lipid rafts may be the intrinsic ability of these lipids to cluster into domains with a close lipid packing as has been shown in biophysical studies (Simons and Ikonen 1997; Rietveld and Simons 1998; Brown and London 2000). The association of cholesterol with the sphingolipids is most likely strengthened by hydrogen bonding between the 3-OH group of the sterol and the amide function of the sphingolipid ceramide backbone (Simons and Ikonen 2000). Phospholipids containing (highly) unsaturated fatty acids are not expected to be in lipid rafts since they would loosen lipid packing and thus inhibit lipid domain formation. In contrast, phospholipids containing saturated fatty acids have the ability of more condensed packing and of promoting domain formation. Therefore, phospholipids with saturated fatty acids are expected to be highly enriched in lipid rafts (Simons and Vaz 2004).

The assumption that rafts from biological membranes are enriched in phospholipids with saturated fatty acids is based on extrapolation from model membranes (Ahmed et al. 1997; Anderson and Jacobson 2002; de Almeida et al. 2003; Scherfeld et al. 2003; Crane and Tamm 2004) but remains to be experimentally validated. However, these model membranes may show an oversimplified picture since mostly binary and ternary lipid mixtures [in most cases, cholesterol, sphingomyelin (SM) and dipalmitoyl phosphatidyl choline] were used in these model systems and the composition of these model membranes do not reflect the large complexity of biological membranes. Moreover, biological membranes contain diverse membrane proteins that may play a role in membrane micro-domain properties.

In this study, we isolated the DRM fraction from different cell types with a cold Triton X-100 method in which cells are treated with 1 % Triton  X-100 at 4 °C and the resulting suspension is placed into a discontinuous sucrose gradient for separating the Triton X-100 soluble and insoluble fractions. The latter floats to a low sucrose density and is defined as the detergent-resistant membrane (DRM), supposed to be enriched with the L_o_ phase of the original lipid rafts. The DRM that appears as an opaque white band in the interphase of 30 and 5 % sucrose in the gradient was processed for negative staining and ultrastructural properties were determined using transmission electron microscopy. Although the question remains whether the DRM fraction resembles the in vivo situation (Simons and Ikonen 1997; Hattersley et al. 2013), we chose to use the Triton X-100 method to make a comparison with most other studies possible. In this respect, an extensive study of Schuck and colleagues on the resistance of cell membranes to different detergents showed that Triton X-100 (used in the present study) and CHAPS are the most reliable detergents for analyzing raft association (Schuck et al. 2003). They concluded that, despite its disadvantages, “detergent isolation remains the starting point for defining membrane subdomains”.

In the current study, the lipid content and composition of extracted lipids from the DRM versus the whole cell were compared. We examined whether the polyunsaturated phospholipid species are indeed excluded from DRMs derived from biological membranes and we determined which specific phospholipid species are enriched in DRMs. We qualitatively and quantitatively analyzed the lipids that are present in the DRM fraction of biological membranes derived from three cell types with considerable differences in total lipid composition (Evans et al. 1980; Lynch et al. 1986; DeLong et al. 1999): Madin-Darby canine kidney (MDCK) cells, McArdle cells and porcine sperm. MDCK cells have a relative saturated lipid composition while McArdle cells are somewhat more unsaturated. The rationale for comparing these cells with sperm is that sperm almost exclusively contains polyunsaturated phospholipids but the presence of lipid rafts in sperm cells has been reported in the literature (Travis et al. 2001; Selvaraj et al. 2009). We chose MDCK cells as a reference as this cell type is established as a model cell line for studying lipid rafts (Verkade et al. 2000; Gallegos et al. 2006). McArdle cells were chosen as an additional comparative cell model since Pike et al. (2002) demonstrated in HeLa cells (cervix-derived epithelial cell line) that the DRM fraction contained substantial amounts of (poly)unsaturated fatty acid containing phospholipids. Beyond the study of cholesterol and phospholipids, the partitioning of glycolipids from sperm and MDCK cells into the Triton soluble and insoluble membrane fraction (representing the L_d_ and L_o_ phase) was also compared. Both cell types are enriched in glycolipids (Gadella et al. 1993; Pescio et al. 2012). In MDCK, the glycolipid fraction exclusively contains ceramides glycosylated predominantly with galactosyl-3-sulfate (sulfatides) while sperm exclusively contains alkyl-acyl-glycerol, which is only glycosylated with galactosyl-3-sulfate (seminolipid; for review, see Vos et al. 1994). McArdle cells were not used for this purpose as they only contain trace amounts of glycolipids.

Implications of the observed differences of DRM versus whole cell lipid composition, with respect to what this may imply for lipid raft composition in terms of membrane fluidity and membrane heterogeneity dynamics, are discussed including the role of cholesterol, cholesterol interacting proteins and glycolipids, with special reference to sperm physiology.

## Materials and methods

### Sperm preparation

Semen was obtained from the Cooperative Centre for Artificial Insemination in Pigs “Utrecht en de Hollanden” (Bunnik, the Netherlands). Semen was filtered through gauze to remove gelatinous material. Sperm cells were washed on a discontinuous Percoll (Amersham Biosciences, Uppsala, Sweden) gradient as described (Flesch et al. 1998). All solutions used were iso-osmotic (285–315 mOsm/kg) and at room temperature unless stated otherwise.

### Cell culture

Madin-Darby canine kidney (MDCK) strain II cells were maintained at 37 °C in 5 % CO_2_ in MEM (Gibco, Paisley, UK) supplemented with 10 % foetal bovine serum (FBS; Gibco) and MEM non-essential amino acids (Gibco). Experiments were performed on confluent or subconfluent cells cultured in 175 cm^2^ culture flasks. McArdle cells (McA-RH7777, ATCC no. CRL-1601) were cultured in Dulbecco’s modified Eagle’s medium (Gibco) supplemented with 10 % FBS and 10 % horse serum (HS; Gibco) and maintained in 175 cm^2^ culture flasks at 37 °C, 5 % CO_2_ under humidified atmosphere.

### Isolation of DRM

DRMs were isolated according to Martens et al. (2000). In brief, washed cells were resuspended in Mes buffer (25 mM Mes, 150 mM NaCl, 1 mM EGTA, pH 6.5, 1 % Triton X-100 that was completed with protease inhibitors: Complete; Roche Diagnostics, Mannheim, Germany) and kept on ice for 30 min. The suspension was mixed with the same volume of an 80 % Mes buffered sucrose solution and a discontinuous sucrose gradient (30 and 5 % sucrose in Mes buffer) was layered on top of it. After 18 h centrifugation (200,000*g*, 4 °C), the gradient was split into 13 fractions of 1 ml. The DRM was isolated as a low-density fraction number 5 and was further investigated on lipid composition. Fractions 1–13 of MDCK cells and sperm were used for detection of glycolipid partitioning in the gradient.

### Dot blotting

For MDCK cells and sperm from the prepared fractions 1–13, the proteins were solubilized with Laemmli buffer and spotted onto a PVDF membrane (*n* = 5). After blocking with 1 % BSA and 0.05 % Tween 20 in TBS (20 mM Tris, 500 mM NaCl; pH 7.4), the spot-blots were incubated with caveolin-1 antibody (LS-A2869; LifeSpan BioSciences, Seattle, WA, USA) 1:2000 in TBS containing 0.1 % BSA and 0.05 % Tween 20. The membranes were washed with TBS containing 0.05 % Tween 20 and subsequently incubated with goat-anti-rabbit-alkaline phosphatase (ECF detection kit; Amersham Biosciences; diluted 1:4000 in TBS containing 0.1 % BSA and 0.05 % Tween 20). Specific antibody binding was detected using ECF substrate (Amersham Biosciences) on a STORM analyzer (Molecular Dynamics, Sunnyvale, CA, USA; van Gestel et al. 2005a). As a negative control, dot blots were made using pre-blocked caveolin-1 antibody (with blocking peptide LS-E28650 according to the manual provided by LifeSpan Biosciences) which did not give any signal above background (data not shown).

### Protein and phospholipid concentration in fraction 13 versus DRM containing fraction 5

The protein concentration of fractions 5 and 13 was determined by the method of Lowry et al. (1951). Lipids were extracted from fraction 5 or whole cells according to the method of Bligh and Dyer (1959). The lipid phosphorous was quantified according to Bartlett (1959).

### Glycolipid DRM partitioning

For MDCK cells and sperm the fractions 1–13 were also used for lipid extraction according to Bligh and Dyer (1959). The total lipid extract was put on an activated silica column on which first the neutral lipids were eluted by chloroform followed by the elution of glycolipids (acetone fraction) (Gadella et al. 1992). The glycolipid fraction was transferred to an HPTLC thin layer chromatography plate and glycolipids were separated and after development of the plate the glycolipids were stained with orcinol to form purple-stained bands (Gadella et al. 1993). Sulfated glycolipids were quantified by the colorimetric assay of Kean (1968) as modified by Radin (1984).

### Negative staining of the DRM

For MDCK, McArdle cells or sperm, 5 μl of fraction 5 was placed on Formvar-carbon-coated copper grid for 30 min. Subsequently, the grids were rinsed three times with PBS and twice with water. Finally, samples were negatively stained by placing the grids for 30 s in 2 % potassium phosphotungstate solution, pH 6.8. The grids were viewed and photographed in a Philips CMI (electron microscope at 100 kV) (see also Vennema et al. 1996).

### Analysis of phosphatidylcholine species

Lipids were extracted according to Bligh and Dyer (1959) from the DRM (fraction 5) and from total cells. From this total lipid extract, sterols and molecular species of phosphatidylcholine (PC) and sphingomyelin (SM) were separated based on Brouwers et al. (1998) with a slightly modified mobile phase of acetonitrile:methanol:triethylamine (25:24:1) on two LiChrospher 100 RP18-e columns (5 μm, 250 × 4.6 mm; Merck, Darmstadt, Germany) in series. Lipids were detected with a Varex MKIII evaporative light scattering detector (ELSD; Alltech, Deerfield, IL, USA) operated at 100 °C at a gas flow of 1.8 l/min. Identification of molecular species was performed by on-line (tandem) mass spectrometry on a API-365 triple stage quadrupole mass spectrometer (Sciex, Ontario, Canada) as described before (Brouwers et al. 1988, 1998). Determination of the position of the ester/ether linkages of aliphatic compounds to glycerophosphocholine was performed according to Vernooij et al. (2002) and Bleijerveld et al. (2007). ELSD data were used for quantification using EzChrom software (Scientific Software, San Ramon, Canada).

### Analysis of phosphatidylethanolamine species

Lipids were extracted according to Bligh and Dyer (1959). Since high amounts of Triton X-100 disturbed the analysis, Triton X-100 was removed with the use of silica columns. Silicagel 60 (Merck) was activated (2 h at 120 °C) and subsequently resuspended in chloroform. A small glass column (∅ 0.5 cm) was packed with this suspension. The lipid extract was dissolved in chloroform:methanol (9:1) and neutral lipids were eluted with 4 column volumes of chloroform, then glycolipids (and Triton  X-100) with 8 column volumes of acetone and finally phospholipids with 4 column volumes of methanol. The fractions were dried under nitrogen and stored at −20 °C.

The obtained phospholipids (methanol fraction) were dissolved in hexane/isopropanol/acetone (82:17:1, v/v/v). Lipid classes were separated on a normal phase column as described in Brouwers et al. (1998) and PE was collected using a flow splitter. PE species were separated on two Synergi 4u MAX-RP 18A columns (250 × 3 mm) (Phenomenex, CA, USA) in series as described in Brouwers and Vernooij (1999) with a slightly modified mobile phase of methanol: acetonitrile (3:2, v/v). Lipids were detected with ELSD and identified with spectrometry as described above. Determination of the position of the ester/ether linkages of aliphatic compounds to glycerolphosphoethanomine was performed according to Brouwers and Vernooij (1999). Quantification was performed as described above for PC species.

### Methyl-β-cyclodextrin incubation

Washed sperm cells were pelleted (10 min, 1000*g*) and subsequently resuspended in Hepes buffer (137 mM NaCl, 20 mM Hepes, 10 mM glucose, 2.5 mM KCl, 10 mg/l kanamycin) containing 0, 1, 2, 5, 10 or 20 mM methyl-β-cyclodextrin (MBCD; Sigma Aldrich Fluka, Zwijndrecht, the Netherlands). Sperm cells were incubated for 30 min at 37 °C (results of sperm experiments have been previously published; Van Gestel et al. 2005b). MDCK cells were washed with PBS and resuspended in serum-free medium containing 0; 1; 2; 5; 10 or 20 mM methyl-β-cyclodextrin (MBCD). The cells were incubated for 30 min at 37 °C and 5 % CO_2_ under a humidified atmosphere. After MBCD treatment, the cells were centrifuged (10 min at 1000*g*), the medium was removed and the pellets were used for isolation of detergent-resistant membrane fractions as described above.

## Results

### Isolation of detergent-resistant membranes

**Fig. 1:**
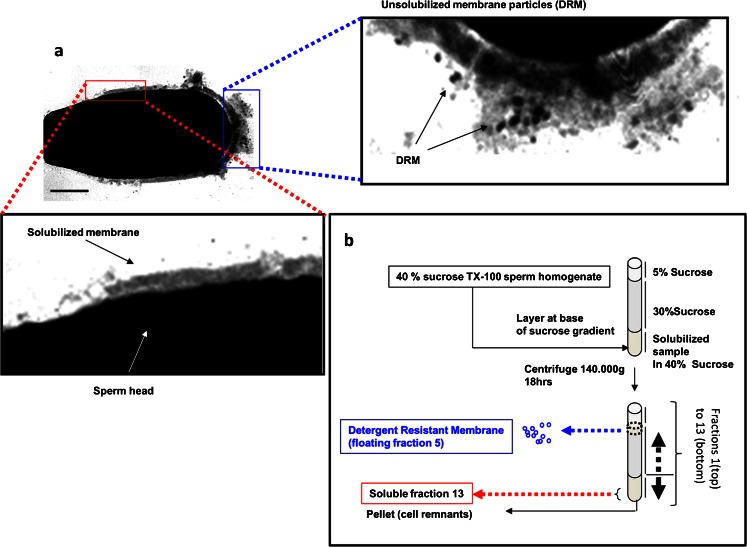
The effect of 1 % Triton X-100 at 4 °C on porcine sperm and a scheme for the separation method used to obtain a soluble and insoluble membrane fraction from sperm, McArdle and MDCK cells. **a** Regional differences of solubilization of sperm membranes after subjection to 1 % Triton X-100 at 4 °C. An ultrathin section of a porcine sperm head visualized with transmission electron microscopy (samples were processed for transmission electron microscopy according to the method of Tsai et al. 2010). *Scale bar* 2 μm. The *red area* (at the equatorial surface area) is shown magnified below and shows that membrane structures are lost due to Triton  X-100 solubilization. The *blue area* (of the apical ridge surface area) is shown magnified on the right (rotated to the right by 90°) and insoluble membrane micro-domains are indicated as DRM. **b** A schematic representation for separating the DRM from the soluble membrane fraction and the cellular remnants

**Fig. 2:**
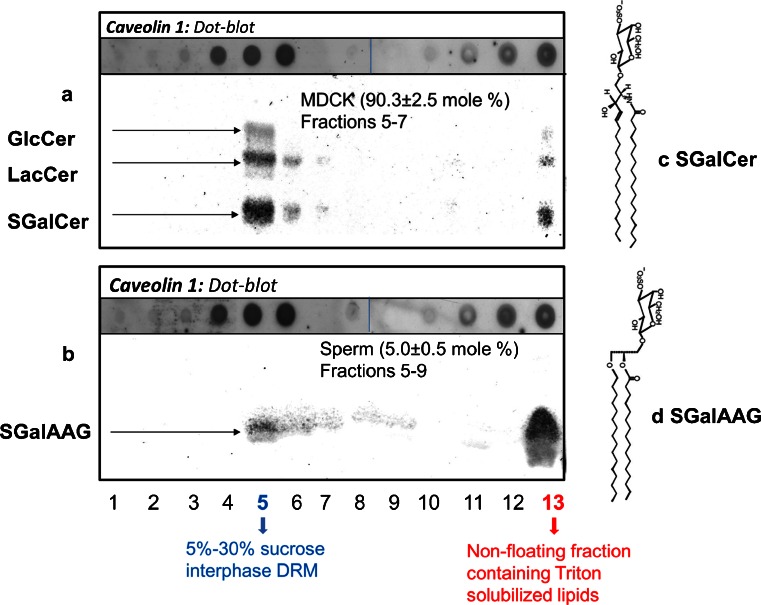
Partitioning of glycolipids and caveolin-1 in the sucrose gradient of 1 % Triton X-100 at 4 °C treated MDCK cells and sperm. The sucrose gradient of MDCK cells and sperm (cf. Fig. 1) was divided into 13 fractions of 1 ml. Proteins of fractions 1–13 were solubilized and transferred to a PVDF membrane (dot blot). Specific antibody binding was detected with enhanced chemifluorescence. For presentation purposes, dots of fractions 9–13 were aligned aside the spots of 1–8; the dots were originally spotted in multiple rows of 8 dots on one PVDF membrane and developed in the same fashion. Lipids from the 1–13 fractions were extracted, from which the glycolipids were purified and spotted on HPTLC plates, which was after development and charred with orcinol to allow purple staining of glycolipids (for method, see Gadella et al. 1993). **a** Dotblot and HPTLC for MDCK cells and **b** for boar sperm cells. The amount of sulfatides (SGalCer for structure: **c** for MDCK and seminolipid; SGalAAG for structure: **d** of fraction 13 versus fraction 5–9) was quantified according to the coloric method of Kean (1968) as modified by Radin (1984). Mean values ± SD are provided (*n* = 5)

We isolated detergent-resistant membrane (DRM) fractions with Triton X-100 and sucrose gradient centrifugation. The DRM fraction appeared as an opalescent band at the 5–30 % density interface of the gradient in all three cell types investigated (the principle is explained for sperm see Fig. 1). To confirm the identity of the opalescent band, we fractionated the gradient and determined the distribution of raft marker protein caveolin-1 in the gradient of sperm and MDCK cells. For porcine and mouse sperm, similar results have been published previously (van Gestel et al. 2005a; Miranda et al. 2009). An enrichment of caveolin-1 was observed in the DRM fraction with a quite similar subfractionation in sperm as observed for MDCK cells (Fig. 2a, b), as well as for McArdle cells (not shown) in agreement with previous reports (Mora et al. 1999). In the DRM fractions, the lipid/protein ratios for MDCK, McArdle and sperm cells were 3.0, 2.3 and 2.9 (nmole lipid phosphate/μg protein), respectively. This is a clear increase compared to the non-DRM fractions (the bottom fraction was considered the non-DRM fraction) of which the ratios were 0.2, 0.07 and 0.14 for MDCK, McArdle and sperm, respectively. This observed enrichment in lipids is in accordance with the literature (Simons and Ikonen 2000; Radeva and Sharom 2004).

### Glycosylated ceramides and glycosylated alkylacylglycerol differ in detergent solubility

Lipid extracts sucrose gradient fractions 1–13 were spotted and developed and stained for quantitation HPTLC plate. Interestingly, only 10 % of the glycolipids of MDCK cells was Triton-solubilized (in fraction 13) and most of the non-solubilized glycolipids appeared in the DRM fraction 5 (with low amounts of incomplete floating glycolipids in fractions 6 and 7) (see Fig. 2a). In contrast, 95 % of the sperm glycolipids was solubilized (fraction 13) while the insoluble glycolipids were also predominantly in the DRM fraction 5 but showed more pronounced amounts of incomplete floating glycolipids in fractions 6–9). (see Fig. 2b). Apparently, glycosylceramides were not soluble for 1 % Triton  X-100 at 4 °C, while sulfogalactosylalkylacylglycerol (also known as seminolipid; Gadella et al. 1992, 1993) was soluble under the same conditions. For structural differences of the most abundant MDCK and sperm glycolipids, see Fig. 2c, d. Note that both lipids have the same galactosyl-3-sulfate head group, another indication that the lipophilic part of glycolipids is determining the DRM preference.

### Ultrastructure of DRM

**Fig. 3:**
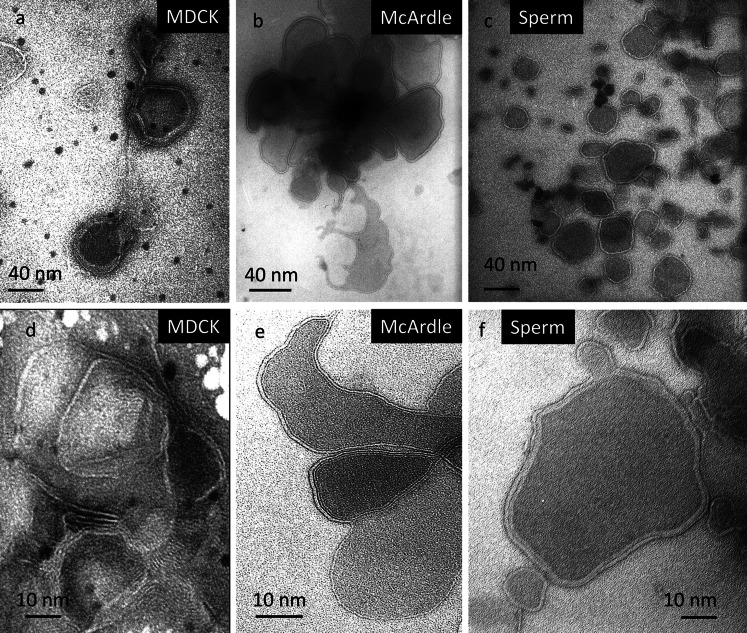
Ultrastructure of DRM derived from MDCK cells, McArdle cells and boar sperm. After subjecting the cells to 1 % Triton  X-100 at 4 °C, the DRM (fraction 5) was isolated as indicated in Fig. 1. A small drop of DRM was spotted on a grid that was processed for negative staining (Vennema et al. 1996) and inspected with transmission electron microscopy

The ultrastructure of the negative-stained (Vennema et al. 1996) DRM fractions derived from MDCK cells, McArdle cells and porcine sperm are depicted in Fig. 3 using transmission electron microscopy. In all three cell types, mono- and multi-lamellar vesicular structures with varying degrees of aggregation were detected. Note that the DRM of MDCK McArdle cells only formed lamellar structures while its neutral lipids were floating on top of the sucrose gradient (fraction 1 data not shown; sperm did not contain neutral lipids). This demonstrates that DRMs indeed contain a high degree of a lipid bilayer preferring and stabilizing lipids but also shows that the appearance of a DRM is different from the native mono-lamellar L_o_ phase domains (lipid rafts) in living cells.

### Phospholipid analysis of the detergent-resistant membrane fraction

**Fig. 4:**
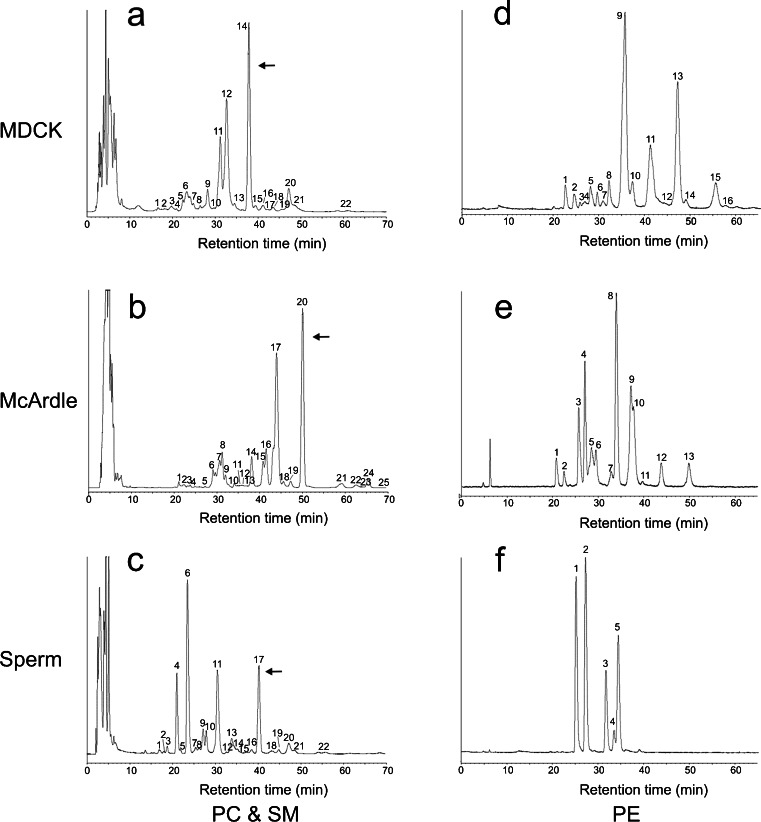
Cell-type specific compositions of lipid molecular species. HPLC chromatograms showing the phospholipid composition as monitored by light scattering detection: **a**–**c** the separation of sterols and molecular species of PC and SM of MDCK cells (**a**), McArdle cells (**b**) and sperm cells (**c**). **d**–**f** The molecular PE species of MDCK cells (**d**), McArdle cells (**e**) and sperm cells (**f**) after preceding isolation of PE by normal phase chromatography. Note that the cell types have a completely different molecular species composition of both PC, SM and PE and that the amount of cholesterol differs as well. *Peak numbers* refer to the identification of the species in Tables 1 and 2. *Arrows* indicate cholesterol

The lipid composition of the DRM fraction 5 was compared to the composition of a total cell extract. To this end, lipid extracts were analyzed by reversed phase HPLC, separating cholesterol, PC and SM molecular species that were subsequently detected and quantified with light scattering detection and identified by on-line tandem MS (Fig. 4). The second most abundant phospholipid class in these membranes, PE, was purified by normal-phase HPLC and molecular species were subsequently resolved with reversed-phase chromatography (Fig. 4). The numbers in Fig. 4 correspond to the molecular species mentioned in Tables 1 and 2. From these data, it is evident that these cell types have very different lipid compositions. In MDCK cells, there is a DRM-specific twofold increase in SM species (Table 1). In sperm cells and McArdle cells, however, this increase is not statistically significant (*P* = 0.16 and 0.07, respectively).

**Table 1:**
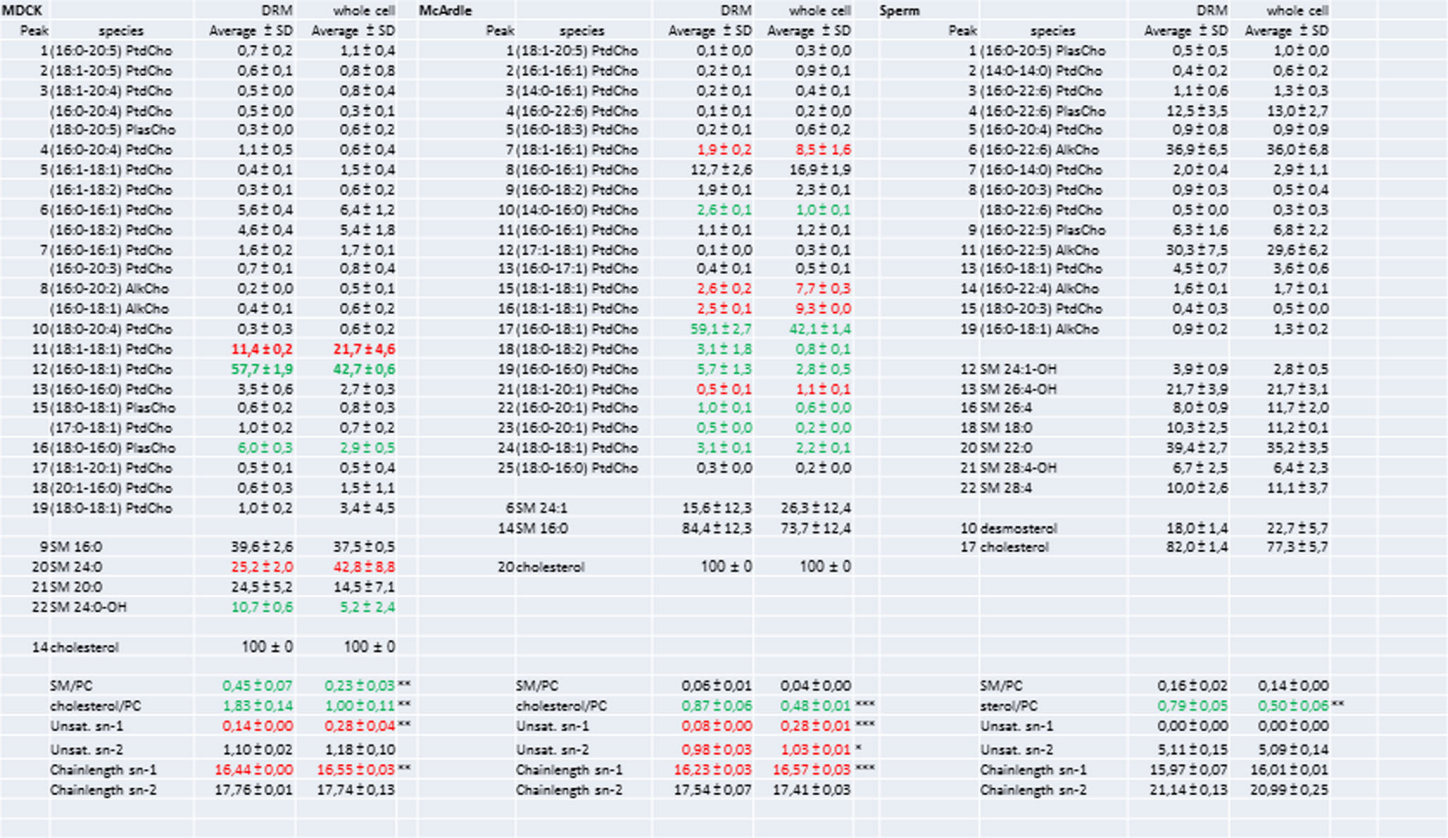
Composition of PC species of the DRM fraction and the total cell extract of MDCK cells (**a**), McArdle cells (**b**) and sperm cells (**c**). Numbers of identified PC species refer to peaks indicated in chromatograms A, B and C of Fig. 6. Mean values expressed in mole% ± SD (*n* = 3) are indicated As with PE species from sperm cells; the relatively high standard deviation in the data was caused by boar-to-boar variation. Within each animal, the composition of DRM and whole cell lipids was nearly identical. The unsaturation and chain length of the *sn-*1 and *sn-*2 substituents are expressed as the average number of double bonds or carbon atoms per fatty acyl residue (taking abundance of species into account). The Cholesterol/PC- and PC/SM ratio are molar ratio’s. Asterisks indicate significant differences between DRMs and whole cell extracts in a Students *t*-test. * *P* < 0.05, ** *P* < 0.01 and *** *P* < 0.001. Red indicates a significant depletion of a lipid component in the DRM when compared to the relative amount detected in whole cells, green indicates a significant accumulation of a lipid component in the DRM when compared to whole cells (for colors *P* < 0.05)

**Table 2:**
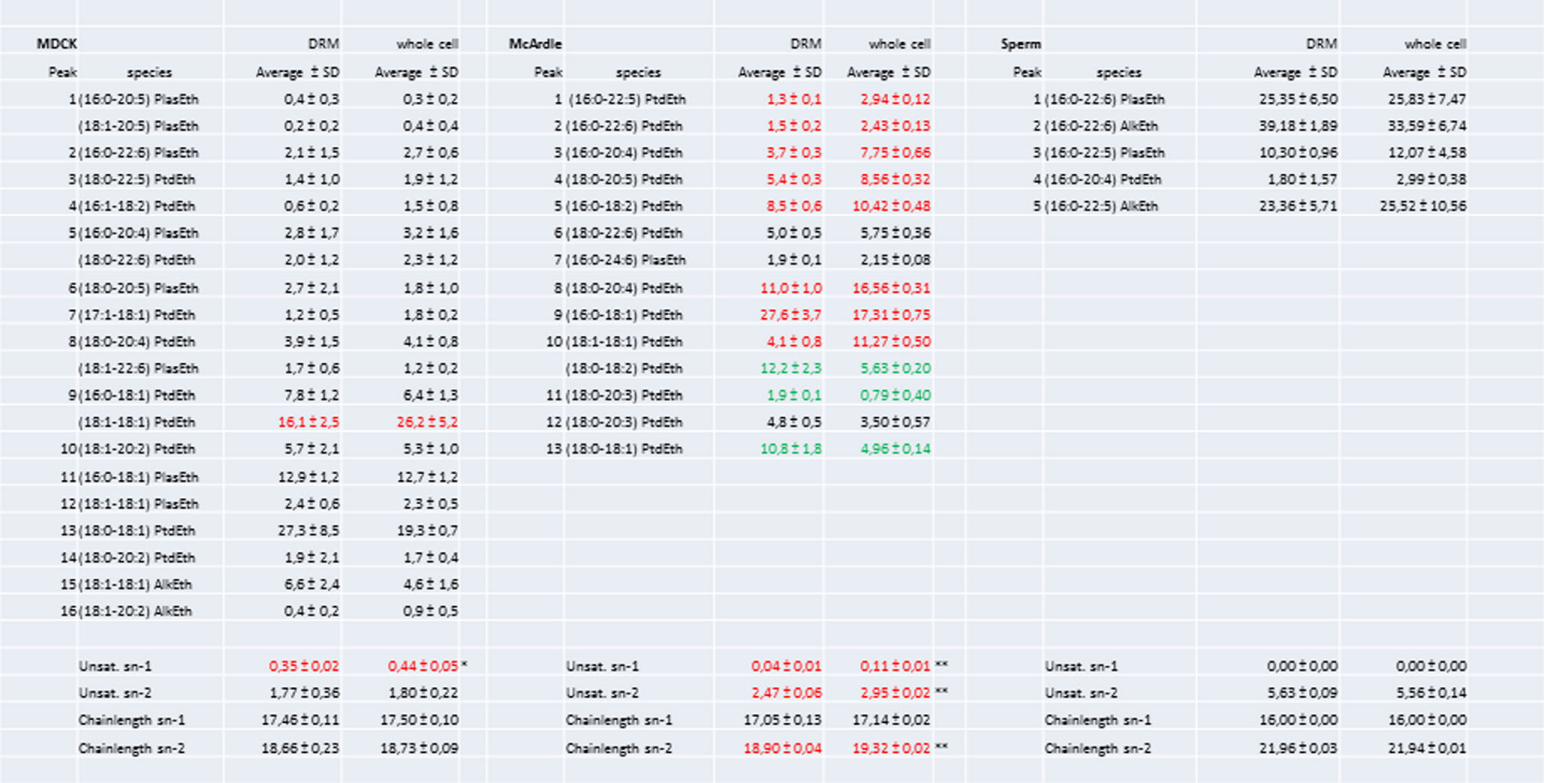
Composition of PE species of the DRM fraction and the total cell extract of MDCK cells (**d**), McArdle cells (**e**) and sperm cells (**f**). Numbers of identified PE species refer to peaks indicated in chromatograms D, E and F of Fig. 6. Mean values expressed in mole% ± SD (*n* = 3) are indicated. The unsaturation and chain length of the *sn-*1 and *sn-*2 substituents are expressed as the average number of double bonds or carbon atoms per fatty acyl residue (taking abundance of species into account). Asterisks indicate significant differences between DRMs and whole cell extracts in a Students *t*-test. * *P* < 0.05, ** *P* < 0.01 and *** *P* < 0.001. Red indicates a significant depletion of a lipid component in the DRM when compared to the relative amount detected in whole cells, green indicates a significant accumulation of a lipid component in the DRM when compared to whole cells (for colors *P* < 0.05)

To circumvent the necessity to compare all individual molecular species in DRM and whole cell extracts, molecular species were classified based on *sn-*1 aliphatic chain length, *sn-*1 aliphatic degree of unsaturation, *sn-*2 aliphatic chain length and *sn-*2 aliphatic unsaturation. Taking into account the relative abundance of the molecular species, an average *sn-*1/*sn-*2 aliphatic degree of unsaturation and chain length was calculated. These averages (and their standard deviations) were subsequently used to detect statistically significant differences between phospholipids from DRMs and whole cells. In MDCK and McArdle cells, in contrast to the *sn*-2 position, the degree of *sn-*1 unsaturation for aliphatic chains both PC and PE differed more substantially (for instance, PC 0.08–0.14 unsaturated carbon bonds in the DRM versus 0.28 unsaturated carbon bonds in the whole cell (Tables 1 and 2); phospholipids derived from DRMs showed significant less unsaturation at the *sn*-1 position than whole cell-derived phospholipids. It should be noted that sperm cell-derived PC and PE only contain saturated *sn-*1 substituents and therefore no difference in *sn-*1 unsaturation was observed in these cells. The degree of *sn-*2 unsaturation was statistically significantly different only in McArdle-derived PC and PE (*p* < 0.05 and *p* < 0.01, respectively) (Table 1). The absolute difference in unsaturation of *sn-*2 PC substituents, however, was very small (0.98 ± 0.03 vs. 1.03 ± 0.01 double bond per substituent). Interestingly, despite the fact that sperm cells were found to contain almost exclusively polyunsaturated *sn-*2 contituents, this did not preclude the isolation of DRMs from these cells.

Small but significant differences in chain length were found for *sn-*1 PC (MDCK and McArdle cells) and *sn-*2 PE (McArdle cells) substituents. In most cases, however, there was no difference in chain length between phospholipids derived from DRMs or whole cells (Tables 1 and 2). No differences were found for PC and PE species with regard to lipid subclasses (diacyl, alkylacyl or alkenylacyl also called plasmalogen) moieties, indicated in Tables 1 and 2 as Ptd, Alk and Plas, respectively.

### Role of cholesterol

The amount of cholesterol present in DRMs differed considerably between the cell types (Table 1; Fig. 2). However, the cholesterol/PC ratio in the DRM fraction compared to the total cell extract was significantly elevated in all cell types investigated; in all DRM fractions, a 60–80 % increase in cholesterol content compared to the total cell extracts was detected. Sperm cells contained a relatively high level of desmosterol, which also increased 60–80 % in the DRM fraction.

**Fig. 5:**
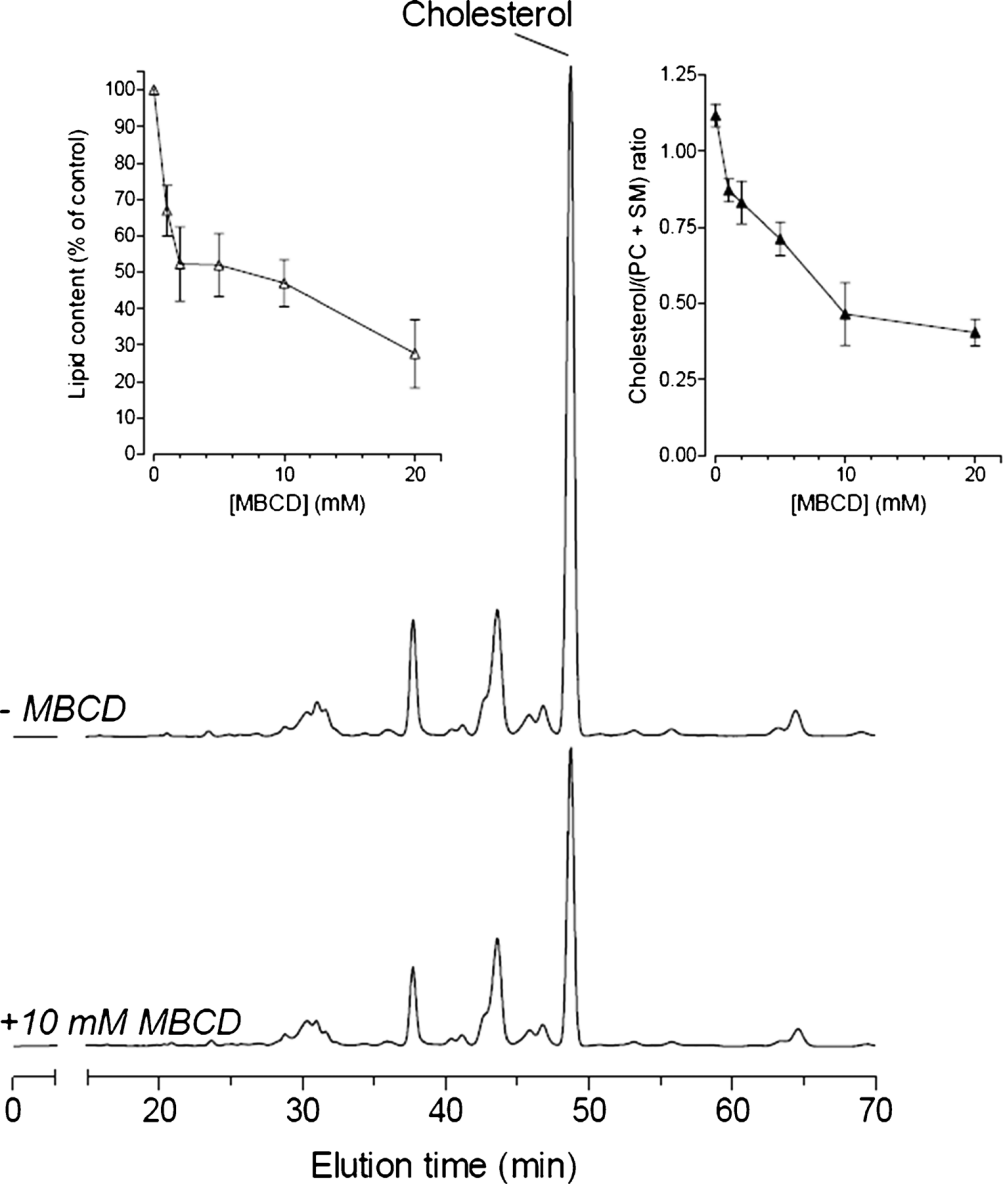
MBCD decreases the amount of lipid in the DRM fraction. HPLC chromatograms showing the separation of molecular PC and SM species and cholesterol of DRM fractions derived from MDCK cells incubated with 0 mM (*top*) and 10 mM MBCD (*bottom*). Note that the PC and SM species composition does not change after MBCD treatment and that cholesterol is specifically extracted from the DRM fraction. Both traces are plotted on the same scale and represent DRMs from an identical amount of cells. The *left inset* shows the decrease in lipids after MBCD treatment and the *right inset *shows the MBCD-mediated, dose-dependent depletion of cholesterol from the DRM fraction. Similar results with porcine sperm have been published previously (van Gestel et al. 2005a)

Since loss of detergent resistance by cholesterol depletion is considered as a characteristic of lipid rafts, MBCD sensitivity has become a hallmark in the study of lipid rafts (Simons and Vaz 2004). We therefore incubated cells with MBCD (with 0, 1, 2, 5, 10 or 20 mM for 30 min). A clear, MBCD-dependent, cholesterol decrease was observed in the DRM fraction without affecting the PC and SM species composition (Fig. 5). At concentrations of MBCD above 10 mM, an overall lipid depletion from the DRMs was observed rather than a specific cholesterol extraction. Even under the most severe cholesterol depletion conditions (incubation with 20 mM MBCD) still PC and SM species could be detected in the DRM fraction and their lipid composition was identical to those observed in DRM from cells not treated with MBCD. For sperm, similar observations have been reported previously; although DRM was more sensitive to MBCD (van Gestel et al. 2005b) this may be the result of a lower cholesterol content in these DRMs compared to MDCK cells in combination with a higher unsaturation index of hydrocarbon tails coupled to the fatty acids (Table **1**).

## Discussion

Evidence for the existence of lipid ordered (L_o_ lipid phase) micro-domains, also called lipid rafts, in living cells is accumulating (Dietrich et al. 2002; Gaus et al. 2003; Pierce 2004; Diaz-Rohrer et al. 2014) but detailed knowledge about the lipids in these domains is lacking. Most lipid-related research in this field has been of a biophysical nature, in which model membranes were used to determine which lipids have the ability to form lipid domains. Those studies showed that a combination of cholesterol, sphingolipids and (phospho)lipids with saturated fatty acid chains are able to spontaneously form microdomains that are detergent-resistant (Ahmed et al. 1997; de Almeida et al. 2003; Scherfeld et al. 2003; Crane and Tamm 2004). However, the work with model membranes has several disadvantages. Obviously, model membranes are a simplification of cellular membranes. In most model systems, binary and ternary lipid mixtures (mainly dipalmitoylPC, cholesterol and SM) are used that do not reflect the complex lipid composition in living cells. Furthermore, the molar percentages of the lipids used in those systems are in general not reflecting those reported for biological membranes. Finally, these model membranes do not take into account that (microdomain) proteins could play a role in domain formation and stability.

### Isolation and structure of detergent-resistant membranes

From all three cell types studied here, a DRM fraction could be isolated using the routinely used cold Triton X-100 method. Western blot analysis revealed a clear enrichment of caveolin-1 in the DRM fraction in MDCK cells and sperm as has been described previously (Scheiffele et al. 1998; van Gestel et al. 2005a). Furthermore, the DRM fractions showed, independent of the cell type, a high lipid/protein ratio compared to the non-DRM fractions. This is in agreement with previous reports in which this high lipid content was suggested to be caused by the tight lipid packing in DRMs (Simons and Ikonen 2000; Brown and London 1998). Together, these characteristics (detergent resistance, caveolin-I enrichment and a high lipid/protein ratio) show that subsequent lipid analyses were performed on standard DRMs. Interestingly, DRM derived from all cell types had a multi-lamellar and mono-lamellar vesicular ultrastructure with a varying degree of aggregation. Altogether, this shows that the floating fraction 5 contained high amounts of a lipid bilayer preferring lipids enrichment of cholesterol and caveolin and a low total amount of membrane proteins. It also demonstrates that DRM has a non-physiological appearance when compared to the L_o_ lipid phase membrane microdomains in the living cell (Lingwood and Simons 2010).

### Ceramide versus alkylacylglcerol as a lipid anchor for glycolipids

Interestingly, under the routine cold Triton X-100 treatment, glycosylceramides of MDCK cells predominantly showed detergent resistant properties in line with Moyano et al. (2014), while sperm seminolipid (a glycosylalkylacylglycerol) was predominantly detergent-soluble. In both cases, the most abundant glycolipid carried a galactosyl-3-sulfate head group. This implies that the lipophilic part of glycolipids determines their partitioning into the L_o_ versus L_d_ phase of lipids and that the sperm seminolipid is only to a low extent represented in the lipid raft. In fact, this finding is supported by Gadella et al. (1994, 1995) who showed that the seminolipid during sperm capacitation is moving out of the area where sperm capacitation-specific raft aggregation (van Gestel et al. 2005a) is taking place. It is proposed that the seminolipid prevents acrosome fusions at the equatorial area of the sperm head (Flesch and Gadella 2000), thus leaving a specific surface area of the sperm head intact. This so-called equatorial segment is the specific site for binding and fusion of the sperm cell with the oocyte’s surface (oolemma) and thus involved in fertilization (Gadella and Evans 2011).

### The extent of sphingomyelin and sterol enrichment in the DRM fraction is cell type dependent

We performed lipid analysis on the DRM (fraction 5) to investigate which characteristics are common to DRMs and which characteristics vary between DRMs of different cell type. We found SM species to be enriched two-fold in the DRM fraction of MDCK cells. In sperm and McArdle cells on the other hand, we observed no statistically significant enrichment of SM. Furthermore, the SM/PC and sterol/PC ratios clearly show that there was no fixed stoichiometry between PC, SM and cholesterol in the DRM fractions of the different cell types. In all cases, a clear enrichment of cholesterol in the DRM was observed when compared to whole cells.

### Phospholipid species containing polyunsaturated sn-2 fatty acid chains are equally distributed between the DRM and non-DRM fraction

Unexpectedly, the DRM fraction of all cell types studied here contained abundant amounts of *sn*-2 polyunsaturated fatty acid containing PC and PE species. In fact, the degree of unsaturation of PE and PC was not different from that of the total cellular lipid fraction, indicating that the *sn*-2 acyl chains of the most abundant phospholipids classes in mammalian membranes do not discriminate between the DRM and the detergent-soluble fraction of those membranes.. These results do not support the data reported by Schuck et al. (2003): They found a lower unsaturation degree in hydrocarbon chains attached to PC in the DRM derived from MDCK cells when compared to whole cell lipid extracts. In the current study, a much more detailed view in lipid composition of DRM has been achieved and, although we also report on a small difference in saturation degree in MDCK cells, this was not due to the *sn*-2 but only to the *sn*-1 position (see next paragraph). Note that in MDCK cells the sn-2 fatty acids chains of PC contain >7.8 more unsaturated carbon in the DRM when compared to *sn*-1 fatty acids, while in the whole cells this ratio was >4.2. Our work on boar sperm cells is very illustrative of the finding that the *sn*-2 fatty acid unsaturation degree does not accumulate in the DRM, as they virtually exclusively contain polyunsaturated fatty acids (with >5 unsaturated carbon bonds per acyl moiety) at the *sn*-2 position of phospholipids. Only less than 3 mol% of total PC and trace amounts of PE carried saturated fatty acids at the *sn*-2 position. There was no enrichment of these saturated PC species in the sperm DRM fractions. This clearly demonstrates that the degree of saturation of the fatty acid on the *sn-*2 position does not determine its presence in the DRM fraction. This is in accordance with previous work (Stulnig et al. 2001; Pike et al. 2002), who also found abundant polyunsaturated lipids in the DRM fraction. The fact that DRMs from sperm can be isolated and that these DRMs predominantly contain polyunsaturated fatty acids is in contrast with the suggestion that Triton X-100 treatment causes artificial clustering of specific (long-chain and saturated fatty acid-containing) phospholipids (Hooper 1999).

### Plasmalogen and alkylacyl phospholipid subclasses do not discriminate between the DRM and non-DRM fraction

Sperm cells are also valuable model cells to detect the distribution of plasmalogen and alkylacyl phospholipids besides the diacyl phospholipids, since these cells contain abundant amounts of all three phospholipids subclasses. No significant differences in the lipid subclasses of both PC and PE could be detected between the sperm DRM and the total lipid fractions. This confirms the work of Pike et al. (2002) who did not find any differences in diacyl and plasmalogen subclasses between DRMs and total membranes using the detergent method. We further extended these observations by demonstrating that the alkylacyl species also showed no preference for DRMs. In contrast, using a non-detergent isolation method for membrane lipid microdomains, Pike et al. did detect enrichment in ethanolamine plasmalogens in lipid rafts. However, unlike the isolation of membrane microdomains using Triton X-100 or CHAPS as detergents, isolation with weaker detergents or without detergents is under considerable debate as they allow co-isolation of proteins and lipids that are normally not present in DRMs (Flesch et al. 1998; Martens et al. 2000; Sampson and Dart 2008) and in sperm result in different subtypes of rafts (Asano et al. 2009)..

### Saturated sn-1 fatty acid chains of phospholipids are enriched in the DRM fraction

A remarkable resemblance between MDCK cells and McArdle cells is the decrease in (18:1–18:1) PtdCho and increase in (16:0–18:1) PtdCho in the DRM fraction. This phenomenon was not observed in sperm cells as these cells did not carry any unsaturated *sn*-1 fatty acid chains on phospholipids. These abundant phospholipids only differ by the fatty acid moiety on the *sn*-1 position, suggesting that this particular fatty acid moiety plays an important role for the localization in lipid rafts. For PE species, the same phenomenon was seen in the most abundant species: in MDCK cells, there is a decrease of (18:1–18:1) PtdEth, while (18:0–18:1) PtdEth is increased in the DRM fraction, while in McArdle cells both (16:0–18:1) PtdEth and (18:0–18:1) PtdEth had increased. This indicates that, at the *sn*-1 position of phospholipids, a 18:1 fatty acid is not, while a 16:0 or 18:0 fatty acid is, favorable for inclusion in DRMs. The data of Stulnig et al. (2001) show a similar enrichment of 16:0 and 18:0 fatty acids in the DRM fraction of Jurkat T cells, while 16:1 and 18:1 fatty acids were excluded from DRMs. Unfortunately, the position of the fatty acids was not determined. One should note that the inclusion or exclusion of particular fatty acids in DRMs is not absolute: phospholipids with a 16:0 or 18:0 *sn*-1 substituent are also found outside DRMs and (mono-)unsaturated *sn*-1 substituents are present in DRMs. In addition to this, all PC and PE species in sperm cells contained saturated fatty acids on the *sn*-1 position, demonstrating that the inclusion of saturated *sn*-1 constituents and the exclusion of unsaturated *sn*-1 constituents are not the driving force for the formation of micro-domains. The requirement for saturation at the *sn*-1 position is not entirely absolute. The McArdle PE fraction contained only a single (abundant) molecular species with an unsaturated *sn-*1 substituent: (18:1–18:1) PtdEth and this species was clearly excluded from the DRM fraction (4.1 ± 0.8 % in DRMs vs. 11.3 ± 0.5 % in whole cell extracts). However, this was not the only excluded molecular species, as several *sn-*1 unsaturated species were also excluded (peaks 1–5 and 8). The exclusion of these latter species, however, was not as pronounced as observed for (18:1–18:1) PtdEth, leading to an overall decrease in *sn-*1 unsaturation, which was highly significant (Table 2, *p* < 0.01). The fact that we found the same DRM specific enrichment of *sn*-1 16:0 and 18:0 in molecular species of PE (which is predominantly present in the inner leaflet of the plasma membrane (Cullis et al. 1996)) suggests that the inner leaflet is also involved in DRM formation according to the same principle. This idea is in agreement with previous work showing protein clustering into micro-domains at the inner leaflet (Prior et al. 2003).

### Chain length does not differ between DRM and whole cells

**Fig. 6:**
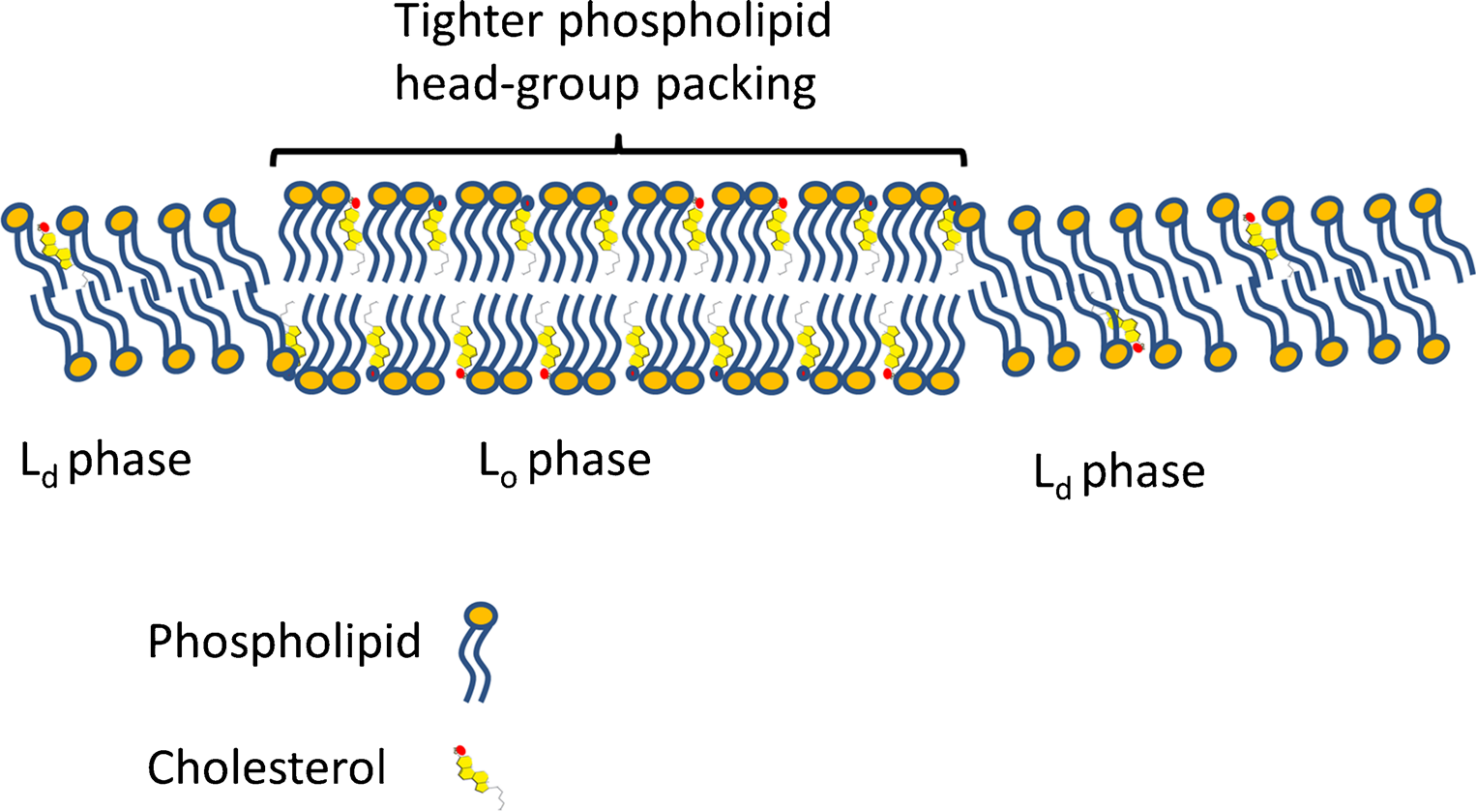
Proposed model for differences between a lipid ordered versus disordered bilayer. The cholesterol-induced tighter ordering of phospholipid head groups is in our model supposed to cause the detergence resistance rather than the hydrophobic fluidness of hydrogen carbon chains attached to the phospholipids. The wider distance of phospholipids in the L_d_ phase in our model allows intercalation of the detergent and this is the solubilization of L_d_ ordered lipids. As is measured in this study, under a threshold concentration of MBCD, lowered cholesterol levels from DRM do not disrupt the DRM and we expect that despite cholesterol removal the phospholipid head groups remain tightly packed. Above a threshold concentration of MBCD, the whole DRM becomes disrupted as phospholipids become disorganized in phospholipid head group packing. For MDCK, this level was reached at >10 mM MBCD while sperm DRMs became disrupted at 2–5 mM (van Gestel et al. 2005b). This difference may be explained by the lower abundance of cholesterol in the DRM of sperm when compared to MDCK, presumably making them more sensitive for cholesterol depletion. In this simplified model, we have not included information on the DRM accumulation of sphingolipids - including the ceramide based sulfatides of MDCK cells and the exclusion of the alkylacyl based seminolipid of sperm; two glycolipids with the same head group (see Fig. 2). This phenomenon shows that the cholesterol induced tighter packing of phospholipid head groups does have an impact on attraction and repulsion of glycolipids. Likewise, the specific attraction of specific membrane proteins and gangliosides is not included in this model

Interestingly, there was little or no difference between the average chain length of phospholipids species in DRMs versus whole cells (Tables 1, 2). Although on some occasions a statistically significant difference was found (e.g., the average length of the *sn*-1 substituent in PC from MDCK cells), it seems unlikely that this very small difference (an average length of 16.44 vs. 16.55 carbon atoms) has any biological implications. An eventual difference in thickness of the lipid bilayer, as is observed for lipid rafts using atomic force microscopy (Saslowsky et al. 2002), can thus not be explained by longer aliphatic chains of phospholipids but could be determined by a higher cholesterol/phospholipid ratio causing a rigidifying or straightening of the unsaturated fatty acid-containing phospholipids and a more up-right orientation of saturated fatty acids containing phospholipids (for model see Fig. 6). In fact, the size of the height differences measured by Saslowsky et al. (2002) may also be an indirect effect of the L_o_ phase of phospholipids: it is known that the lipid raft (and the DRM derived from cells) are enriched in GPI-anchored proteins as well as membrane proteins with longer transmembrane-spanning alpha helices and gangliosides. These structures may on their own behalf interact with specific glycocalyx components that altogether can cause the noted height differences as reported by Saslowsky et al. (2002).

### Role of cholesterol in raft stability

Cholesterol was enriched in all DRM fractions compared to the corresponding whole cell extracts (cf. Pike et al. 2005). It is important to note that a highly different mole % of cholesterol was found in the DRM fractions isolated from different cell types as well as in whole lipid extracts from the three cell types. In all three cell types, an approximately 70 % increase of cholesterol content was found in the DRM fraction compared to the total membrane. The total cell extract used in this study contains all lipids, including the DRM-specific lipids. Therefore, the above-mentioned differences between DRM and total cell extract will underestimate the differences between DRMs and non-DRM fractions. Desmosterol, the last intermediate in cholesterol biosynthesis, is present in high amounts in sperm cells (Brouwers et al. 2011; Boerke et al. 2013). Desmosterol showed a similar partition behavior as cholesterol, probably since the only difference between cholesterol and desmosterol is one double bond at position 25. This double bond apparently has little to no effect on the postulated interaction of the sterol with the fatty acid chains or on the potential hydrogen-bond formation with the ceramide group of the sphingolipids (Brown 1998). Here, we show for the first time that desmosterol belongs to the group of DRM preferring sterols. This extends the work of Xu et al. (2001), who showed that natural sterols other than cholesterol also have domain-promoting activity.

MBCD-induced cholesterol depletion and L_o_ phase disintegration into the L_d_ phase of lipids are important criteria for lipid rafts (Simons and Vaz 2004). Therefore, we examined the effects of MBCD on the lipid composition of DRMs. DRMs obtained from cells that were incubated with low levels of MBCD showed a specific decrease in cholesterol levels. At high MBCD concentrations, a general loss of sterols, PC and SM from the DRM fraction was observed (this study; van Gestel et al. 2005b). This indicates that: (1) DRMs dissociate (as reflected by PC and SM solubilization) but only above a certain threshold of cholesterol depletion; and (2) in the DRM, PC and SM molecular species compositions are independent of cholesterol concentration as the PC and SM compositions remain unchanged even at the highest MBCD concentration. Taken together, this indicates that much lower amounts of cholesterol than actually present in DRMs are sufficient to keep membrane areas detergent-resistant. This observation may underlie the big differences in the cholesterol levels of the DRM fraction between the three cell types. However, one has to be cautious with interpreting these data as, in each cell, MBCD will only extract cholesterol from the exoplasmic half of the lipid bilayer from the plasma membrane. A certain proportion of the L_o_ phase preferring lipids is either at the cytoplasmic site of the lipid bilayer from the plasma membrane or located in intracellular organelles and thus is hidden for MBCD. This may partly explain the invariance of phospholipid composition in the DRM and the incomplete removal of cholesterol after MBCD treatment.

## Conclusions

We analyzed the lipid composition of DRMs from three mammalian cell types. The lipid composition of DRMs was highly cell-type-dependent. There is no “magical” raft mixture but rafts can have multiple compositions dependent on the cell type (de Almeida et al. 2003). These various compositions may reflect the differences in size, topology and dynamics of lipid rafts in living cells. In general, the DRMs were enriched in sterols and did show a significant decrease in *sn*-1 but not *sn*-2 unsaturated phospholipids. Plasmalogen and alkylacyl phospholipids showed no preference to DRMs versus detergent soluble membrane areas. Finally, depletion of cholesterol left the phospholipid content in DRMs largely unaffected, although severe cholesterol depletion led to phospholipid solubilization. Taken together, our data indicate that the now-existing models for lipid rafts have to be refined, since many models assume that lipid–lipid interactions between saturated phospholipids and, for instance, cholesterol are the driving force for the constitution of DRMs (Simons and Vaz 2004). The fact that DRMs can be isolated from sperm cells makes it unlikely that cholesterol interaction with saturated phospholipids is the driving force for DRM formation or stabilization (Wang and Silvius 2001). In view of our data, we suggest that a specific interaction of cholesterol, sphingolipids and cholesterol binding proteins may be the basis of L_o_ lipid phase formation in which a relatively passive pool of phospholipids becomes higher ordered or rigidified. Once formed, these lipid rafts have been sufficiently stabilized to become resistant to the cold Triton X-100 detergent conditions in contrast to the remaining L_d_ lipid phase (for model, see Fig. 6 and the computer simulations reported by Róg and Vattulainen 2014). The resulting DRM can keep its L_o_ phase properties even after MBCD-induced extraction of a portion of its cholesterol; only at high MBCD concentrations are the cholesterol levels so much depleted that the detergent resistance is not further maintained and the DRM is disrupted.

### Implications for sperm physiology and sperm-zona pelucida and/or cumulus interactions

The findings described in this paper imply that the sperm L_o_ lipid phase has quite dynamic properties that may underlay lateral regionalization features of sperm surface and the capacitation-dependent reorganization thereof. During sperm capacitation, a reverse cholesterol transport is activated resulting in the loss of 30–40 % of its surface cholesterol to albumin (in vitro) or high-density lipoprotein complexes (in vivo) (Flesch et al. 2001; Leahy and Gadella 2015). Importantly, in vitro capacitation of sperm in the presence of albumin did not result in cholesterol depletion in the DRM fraction (in contrast to MBCD treatment; van Gestel et al. 2005b). Nevertheless, MBCD treatment allows cholesterol depletion in sperm and this does result in increased zona binding of stallion sperm (Bromfield and Nixon 2013a, b) and to some extent in vitro fertilization of porcine oocytes (Boerke et al. 2013). Note that sperm capacitation is a process essential for sperm to become competent to fertilize the oocyte (Gadella et al. 2008; Aitken and Nixon 2013). The apparent non-DRM cholesterol depletion (Leahy and Gadella 2015) is likely involved in the typical aggregation of lipid rafts in capacitating sperm (van Gestel et al. 2005a). This raft aggregation is induced after the capacitation-induced protein kinase A activity that caused the raft-modulating protein lipocalin 2 (Lingwood 2014) to bind to raft PE (Watanabe et al. 2014). For porcine sperm, caveolin-rich raft-aggregated areas emerge at its surface involved in binding to the extracellular vestments of the oocyte (cumulus layer and the zona pellucida). Both the DRM of sperm as well as this apical sperm head surface are rafts that aggregate and are extremely highly enriched in zona pellucida and/or cumulus binding protein complexes (van Gestel et al. 2005a; 2007; Nixon and Aitken 2009; Nixon et al. 2009; 2011; Reid et al. 2011; Bromfield and Nixon 2013a, 2013b; Caballero et al. 2012; Jones et al. 2010; Tanphaichitr et al. 2007). The same surface area contains SNARE proteins (Tsai et al. 2007), which are involved in the docking of the acrosome during sperm capacitation (Ackermann et al. 2008; Tsai et al. 2010, 2012; Zitranski et al. 2010). Interestingly, the association of CDC42 and the oligomerization of caveolin-1 have been described (Baltiérrez-Hoyos et al. 2012) and also, the involvement of CDC42 in lipid raft-mediated synaptic delivery has recently been reported (Brachet et al. 2015). These, together with the recruitment of Rab 3A to the plasma membrane (Belmonte et al. 2005) and the finding that phospholipase B is activated after reverse cholesterol transport from murine sperm (Asano et al. 2013a, b) are all preparative steps for the acrosome reaction that deserves more research attention. The capacitation-dependent formation of zona pellucida and/or cumulus binding protein complexes as well as the docking of the acrosome make sperm prepared to correctly interact with the cumulus and zona pellucida. Firstly, the cumulus/zona pellucida binding affinity is generated by this process and secondly, the docked acrosome is provoked to induce multiple fusions with the sperm plasma membrane after the cumulus or zona-induced membrane hyperpolarization and subsequent Ca^2+^, which allows configuration of trans to cis SNARE complexes, allowing the secretion of acrosome contents and thus the penetration of the sperm through the zona pellucida. Of special interest is the notion that the bovine sperm DRM fraction is enriched in Ca^2+^ ATPase (Post et al. 2010). Also relevant in this context is the attraction of endocannabinoid system receptors cannabinoid receptor type 1 (CBR1) and transient receptor potential cation channel 1 (TRPV1) into the DRM of capacitated boar sperm (Botto et al. 2010).

Altogether, this demonstrates that, although DRMs may be created artificially under certain detergent and temperature conditions, they do represent a cholesterol-enriched membrane subfraction that is functionally involved in cellular processes such as mammalian fertilization. For McArdle, MDCK and other cells, their DRM preparations may also be highly enriched with molecules involved in specific cellular processes.
